# Safety Outcomes During Pediatric GH Therapy: Final Results From the Prospective GeNeSIS Observational Program

**DOI:** 10.1210/jc.2018-01189

**Published:** 2018-09-13

**Authors:** Christopher J Child, Alan G Zimmermann, George P Chrousos, Elisabeth Cummings, Cheri L Deal, Tomonobu Hasegawa, Nan Jia, Sarah Lawrence, Agnès Linglart, Sandro Loche, Mohamad Maghnie, Jacobo Pérez Sánchez, Michel Polak, Barbara Predieri, Annette Richter-Unruh, Ron G Rosenfeld, Diego Yeste, Tohru Yorifuji, Werner F Blum

**Affiliations:** 1 Eli Lilly and Company, Windlesham, Surrey, United Kingdom; 2 Eli Lilly and Company, Indianapolis, Indiana; 3 National and Kapodistrian University of Athens, School of Medicine, Athens, Greece; 4 Dalhousie University/IWK Health Centre, Halifax, Nova Scotia, Canada; 5 University of Montreal and CHU Ste-Justine, Montreal, Quebec, Canada; 6 Keio University School of Medicine, Shinjuku-ku, Tokyo, Japan; 7 Children’s Hospital of Eastern Ontario, Ottawa, Ontario, Canada; 8 Hôpital Bicêtre Paris Sud, Paris, France; 9 Ospedale Pediatrico Microcitemico “A. Cao,” AO Brotzu, Cagliari, Italy; 10 Istituto Giannina Gaslini, University of Genova, Genoa, Italy; 11 Corporació Sanitària Parc Taulí, Sabadell, Spain; 12 Hôpital Universitaire Necker Enfants Malades and Université Paris Descartes, Centre des Maladies Endocrines Rares de la Croissance, Paris, France; 13 University of Modena and Reggio Emilia, Modena, Italy; 14 University Children’s Hospital, Bochum, Germany; 15 Oregon Health and Science University, Portland, Oregon; 16 Hospital Vall d’Hebron, Universidad Autónoma de Barcelona, Barcelona, Spain; 17 Osaka City General Hospital, Miyakojima-ku, Osaka, Japan; 18 University of Giessen, Giessen, Germany

## Abstract

**Context:**

Safety concerns have been raised regarding premature mortality, diabetes, neoplasia, and cerebrovascular disease in association with GH therapy.

**Objective:**

To assess incidence of key safety outcomes.

**Design:**

Prospective, multinational, observational study (1999 to 2015).

**Setting:**

A total of 22,311 GH-treated children from 827 investigative sites in 30 countries.

**Patients:**

Children with growth disorders.

**Interventions:**

GH treatment.

**Main outcome measures:**

Standardized mortality ratio (SMR) and standardized incidence ratio (SIR) with 95% CIs for mortality, diabetes, and primary cancer using general population registries.

**Results:**

Predominant short stature diagnoses were GH deficiency (63%), idiopathic short stature (13%), and Turner syndrome (8%), with mean ± SD follow-up of 4.2 ± 3.2 years (∼92,000 person-years [PY]). Forty-two deaths occurred in patients with follow-up, with an SMR (95% CI) of 0.61 (0.44, 0.82); the SMR was elevated for patients with cancer-related organic GH deficiency [5.87 (3.21, 9.85)]. Based on 18 cases, type 2 diabetes mellitus (T2DM) risk was elevated [SIR: 3.77 (2.24, 5.96)], but 72% had risk factors. In patients without cancer history, 14 primary cancers were observed [SIR: 0.71 (0.39, 1.20)]. Second neoplasms occurred in 31 of 622 cancer survivors [5.0%; 10.7 (7.5, 15.2) cases/1000 PY] and intracranial tumor recurrences in 67 of 823 tumor survivors [8.1%; 16.9 (13.3, 21.5) cases/1000 PY]. All three hemorrhagic stroke cases had risk factors.

**Conclusions:**

GeNeSIS (Genetics and Neuroendocrinology of Short Stature International Study) data support the favorable safety profile of pediatric GH treatment. Overall risk of death or primary cancer was not elevated in GH-treated children, and no hemorrhagic strokes occurred in patients without risk factors. T2DM incidence was elevated compared with the general population, but most cases had diabetes risk factors.

## Teaser

Safety of GH therapy was assessed in a pediatric observational study. Death and primary cancer rates were not higher than in the general population; T2DM rate was higher owing to risk factors.

Pediatric GH treatment is approved for several conditions that result in short stature and/or growth failure. Although the most frequent diagnosis is growth hormone deficiency (GHD), other currently approved indications include Turner syndrome (TS), short stature homeobox-containing gene (*SHOX*) deficiency (SHOX-D), Noonan syndrome, Prader-Willi syndrome, growth failure associated with chronic renal insufficiency (CRI), short stature in children born small for gestational age (SGA) who fail to demonstrate catch-up growth by age 2 to 4 years, and idiopathic short stature (ISS). GH therapy is generally considered safe, and serious adverse reactions are infrequent (1–3). However, long-standing concerns exist regarding a potential association between GH treatment and the development or recurrence of neoplasms (4–8) and the effect on glucose homeostasis, including development of type 2 diabetes mellitus (T2DM) (9). More recently, data from the French cohort of the Safety and Appropriateness of Growth hormone treatments in Europe (SAGhE) study raised concerns for premature mortality (10) and intracranial hemorrhage (11) in young adults treated with GH during childhood.

In 1999, GeNeSIS (the Genetics and Neuroendocrinology of Short Stature International Study) was implemented to monitor the safety and effectiveness of GH in pediatric patients. Because rare outcomes are unlikely to be observed in numbers appropriate for statistical analysis in clinical trials, large postmarketing studies, such as GeNeSIS, have been critical in establishing the GH safety profile. The primary safety objectives of GeNeSIS were to determine the incidence of T2DM and primary cancer in GH-treated children. Secondary objectives included characterization of neoplastic disease, especially neoplasm recurrence/progression or second neoplasm (SN) development. In response to the concerns raised by the French SAGhE findings (10, 11), assessments of mortality and stroke were also considered key analyses.

## Patients and Methods

### Patients

Patient data were collected in the prospective, multinational, open-label, GeNeSIS observational program (ClinicalTrials.gov: NCT01088412) for GH-treated pediatric patients; in addition, non‒GH-treated patients with a history of neoplasia or SHOX-deficient diagnosis were allowed. All investigational and treatment decisions were at the discretion of the investigator, and no specific tests or procedures were required before or during study participation.

Reporting of all adverse events (AEs) was required, irrespective of whether a causal relationship with GH was suspected. The study was approved by local ethics committees and was conducted according to the principles of the Declaration of Helsinki, and all applicable country-specific regulatory requirements were followed. Written parental (or guardian) consent for data collection, electronic processing, and publication was provided in accordance with national requirements.

**Figure 1.**
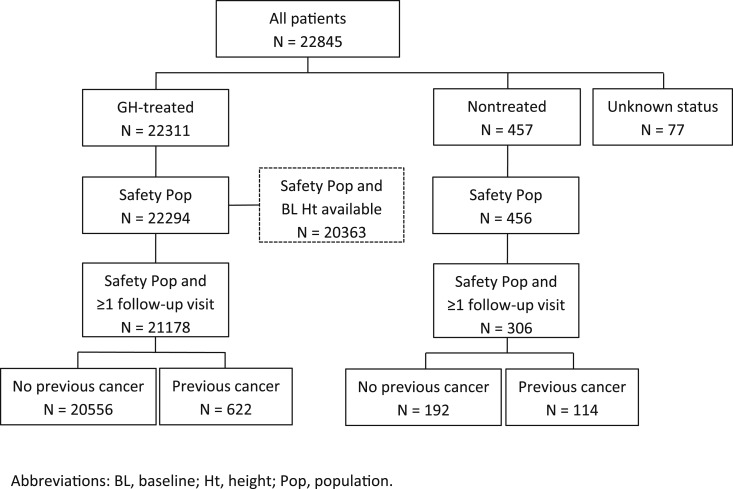
GeNeSIS patient disposition and main analysis populations.

At study closure (data received from March 1999 to September 2015), enrollment had reached 22,845 patients from 827 sites in 30 countries (Supplemental Table 1). Of these, 22,311 patients were treated with GH, 457 were untreated, and 77 had unknown treatment status (Fig. 1). Different populations analyzed included the Safety Population (date of birth/GH treatment status available; N = 22,294), the Safety Population with at least one follow-up visit (N = 21,178), the Safety Population with at least one follow-up visit and no previous cancer (N = 20,556), and the Safety Population with baseline height SD score available (N = 20,363).

### Ascertainment of patient histories and incident cases

Serious AEs (SAEs) that resulted in death, hospitalization, persistent or major disability, or congenital abnormality in offspring or that the investigator considered life-threatening or major were assessed in the overall Safety Population. Treatment-emergent AEs (TEAEs), which first occurred or worsened in severity after GH therapy, were assessed in patients who had at least one postbaseline follow-up visit. All AEs were categorized according to the Medical Dictionary for Regulatory Activities version 18.1.

Treatment-group comparison was inappropriate because the small untreated group was represented primarily by patients with a history of neoplasm or SHOX-D. For outcomes of death, diabetes, and primary cancer, incidence was compared with rates in general population registries, with calculation of standardized mortality ratios (SMRs) and standardized incidence ratios (SIRs). Previous history and incident cases of neoplasia during the study were ascertained by detailed review of study database modules (12), including historical diagnoses, disease-specific check boxes at each study visit, AEs/preexisting conditions, and the Neoplasia Substudy (designed to follow up on the course and treatment of neoplastic disease), with cross-reference to SAE reports in the sponsor’s pharmacovigilance database. Historical factors and causes of death were ascertained using the same information sources, with case-specific details reported previously (13). Histories and incident cases related to abnormal glucose metabolism were ascertained essentially as described for neoplasia and in a previous interim analysis (14), with information from a variety of data sources prioritized according to the scheme presented as Supplemental Fig. 1 and assessment against standard diagnostic criteria (15).

### Statistics

Analyses were conducted using SAS^®^ 9.1. (SAS Institute Inc., Cary, NC) SMRs/SIRs and associated 95% CIs for mortality, diabetes, and primary cancer were determined by country as the ratio between the number of cases observed in GeNeSIS and the expected number of incident cases from general population registries, chosen on the basis of the availability of country-specific data, age-range stratification, and contemporaneousness with the period of GeNeSIS data collection. For mortality statistics, sex-, age-, and calendar year‒specific data from the Centers for Disease Control and Prevention (16) were used for the United States and national or regional data from the World Health Organization (17) were used for all other countries. For diabetes, age-specific and race-specific data (2002 to 2005) from the US SEARCH for Diabetes in Youth Study (18) were used for comparison with all GeNeSIS countries because, to our knowledge, they provide the best reference population for US patients and a finer stratification of ages than pediatric diabetes reference data from countries outside the United States. For cancer rates, sex-, race-, age-, and calendar year‒specific data from the Surveillance, Epidemiology, and End Results Program (19) were used for the United States, and country-, sex-, and age-specific 2012 data from GLOBOCAN (20) were used for all other countries. Country-specific SIRs/SMRs were calculated from the sum of the available sex-, race/ethnicity-, age-, and calendar year‒specific strata and overall from aggregation of the country-specific data. The observed number of cases was assumed to follow a Poisson distribution, and 95% CIs were calculated using an exact method (21).

For all outcomes except diabetes, follow-up time per patient was calculated from the date of first GH dose in GeNeSIS until the date of the last contact (the latest of last follow-up visit, study summary date, cancer onset date, or date of death). For diabetes, follow-up time was calculated from the date of first GH dose because accurate capture of cases that occurred prestudy but after GH initiation was possible.

## Results

### Patient demographics

Of the 22,311 GH-treated patients, the most common diagnostic group was GHD (63%), with the majority having idiopathic GHD (IGHD; 79%). Of patients with organic GHD (OGHD; 21%), the majority had disease due to congenital causes (64%) such as abnormalities in pituitary development. GHD due to acquired causes such as an intracranial tumor and/or its treatment accounted for 36%; the most common intracranial tumors were craniopharyngiomas (N = 271) and medulloblastomas (N = 218). The most common non-GHD causes of short stature were ISS (13%), TS (8%), and being born SGA (6%).

Baseline characteristics and GH treatment parameters for GH-treated patients are shown in Table 1. For all diagnostic groups combined, 60% were male, mean age at GH initiation was 9.7 years, and age at study entry was almost 1 year later (10.5 years), reflecting patients who began GH therapy before study enrollment (∼one-third of the study population). Mean duration of follow-up in the study was 4.2 years, whereas mean duration of treatment was 4.9 years, providing ∼92,000 person-years (PY) of follow-up in the study and ∼104,000 PY of GH exposure.

**Table 1.** Baseline Demographic and GH Treatment Data for the Safety Population, Grouped According to Reported Diagnosis

N*^a^*	Sex (%) F/M	Age at GH Start (y)	Bone Age (y)	Height SDS	Height SDS − Target Height SDS	BMI SDS	Max GH Peak (μg/L)*^b^*	GH Dose (mg/kg/wk)*^c^*
								
20,363	40/60	9.7 ± 3.8	8.5 ± 3.8	−2.5 ± 1.0	−2.0 ± 1.2	−0.4 ± 1.7	7.6 (4.4, 12.1)	0.27 ± 0.10
12,734	33/67	9.7 ± 3.9	8.3 ± 3.8	−2.4 ± 1.1	−2.0 ± 1.2	−0.3 ± 1.6	6.3 (3.6, 9.1)	0.24 ± 0.10
10,189	33/67	10.1 ± 3.6	8.5 ± 3.7	−2.4 ± 1.0	−1.9 ± 1.1	−0.4 ± 1.6	6.8 (4.4, 9.5)	0.25 ± 0.10
2508	35/65	8.2 ± 4.6	7.3 ± 4.2	−2.4 ± 1.5	−2.4 ± 1.5	0.0 ± 1.9	2.9 (1.0, 6.0)	0.23 ± 0.09
2657	28/72	11.3 ± 3.1	10.1 ± 3.2	−2.4 ± 0.8	−1.9 ± 1.0	−0.6 ± 1.4	15.0 (11.6, 20.0)	0.33 ± 0.09
1209	45/55	8.2 ± 3.6	7.1 ± 3.7	−2.7 ± 0.9	−2.1 ± 1.2	−1.4 ± 1.8	11.6 (7.8, 17.5)	0.24 ± 0.10
1712	99/1*^e^*	8.8 ± 3.8	8.2 ± 3.4	−2.6 ± 0.9	−2.6 ± 1.1	0.3 ± 1.5	11.0 (6.6, 16.8)	0.32 ± 0.09
547	58/42	9.3 ± 3.2	8.7 ± 3.2	−2.3 ± 0.8	−1.4 ± 1.0	0.1 ± 1.4	11.6 (8.0, 17.5)	0.30 ± 0.09
82	33/67	8.4 ± 3.9	6.7 ± 3.6	−2.6 ± 1.0	−2.3 ± 1.0	−0.6 ± 1.9	15.2 (11.9, 23.7)	0.30 ± 0.10
1124	40/60	9.4 ± 4.0	8.8 ± 3.8	−2.7 ± 1.3	−2.4 ± 1.5	−0.4 ± 2.0	9.8 (6.0, 16.1)	0.28 ± 0.10

Data are presented as mean ± SD unless stated otherwise.

Abbreviations: BMI, body mass index; F, female; M, male; Max, maximum; N, number; SDS, standard deviation score.

^a^ Maximum N, lower for certain variables.

^b^ Median (Q1, Q3); N (%) with reported GH peak: GHD, 9945 (78); ISS, 2032 (76); SGA, 586 (48); TS, 317 (19); SHOX-D, 175 (32); CRI, 16 (20); and Other, 676 (60).

^c^ Dose at initiation of GH therapy.

^d^ Includes 298 patients with unknown diagnosis.

^e^ Unclear whether male patients with reported TS diagnosis reflect data entry error or so-called “male TS” (*i.e.*, Noonan syndrome).

^f^ Short stature–related diagnoses not in the previous categories, including genetic and cytogenetic conditions, clinical syndromes, skeletal dysplasias, and other non-GHD disturbances of the GH/IGF-I axis.

### General safety outcomes

Table 2 summarizes patients assessed for incidence of key events, cases observed during GeNeSIS, and crude incidence rates. Details on specific outcomes are provided in subsequent subsections. Of the 22,294 patients in the Safety Population, SAEs were reported for 567 (2.5%), with 82 (14.5%) assessed as causally related to GH (Supplemental Table 2). The most prevalent SAEs typically involved childhood infections and conditions associated with short stature diagnoses that may evolve during GH treatment. However, the second-most prevalent SAE was craniopharyngioma with 20 cases, the majority being recurrences. The most prevalent TEAEs were, in general, common childhood ailments, conditions associated with underlying diagnoses, or events previously associated with GH exposure. Overall, 6365 patients (30%; Safety Population with at least one follow-up visit) had at least one TEAE during the study (Table 3), with the highest rate in patients with CRI (49%) and OGHD (47%). With a total of 16,135 separate event episodes, the overall crude incidence rate of TEAE was 175.3/1000 PY.

**Table 2.** Main Study Safety Outcomes in GH-Treated Patients With ≥1 Follow-Up Visit During Study

Outcome Measure	Population at Risk	Patients at Risk (N)	Patients Affected [N (%)]	Crude Incidence (95% CI/1000 PY)^*a*^
Death	All	21,178	42 (0.20)^*b*^	0.46 (0.34–0.62)
Stroke (all)	All	21,178	16 (0.08)	0.17 (0.11–0.28)
Stroke (unknown)	All	21,178	3 (0.01)	0.03 (0.01–0.10)
Hemorrhagic	All	21,178	3 (0.01)	0.03 (0.01–0.10)
Ischemic	All	21,178	10 (0.05)	0.11 (0.06–0.20)
Type 2 diabetes mellitus	All	21,178	18 (0.08)	0.20 (0.12–0.31)
Primary cancer	No cancer history	20,556	14 (0.07)	0.16 (0.09–0.27)
Second neoplasm	History of cancer	622	31 (4.98)	10.69 (7.51–15.19)
ICT recurrence	History of ICT	823	67 (8.14)	16.90 (13.30–21.47)
CP recurrence	History of CP	271	37 (13.65)	26.32 (19.07–36.32)
MB recurrence	History of MB	218	6 (2.75)	5.69 (2.09–12.40)

Abbreviations: CP, craniopharyngioma; ICT, intracranial tumor; MB, medulloblastoma; N, number.

^a^ Reflects first episode of event per patient (where relevant).

^b^ Three additional deaths were reported but not included in calculations because of lack of on-study follow-up visit.

**Table 3.** Frequency of TEAEs by MedDRA Preferred Term Occurring in ≥1.0% of All GH-Treated Patients, Split by Main Short Stature Diagnosis

	All^*a*^	GHD	IGHD	OGHD^*b*^	ISS	TS^*c*^	SHOX-D	SGA	CRI^*d*^	Other
N, %	21,178	13,329	10,450	2831	2686	1791	564	1227	77	1169
Patients with no TEAE	14,813	9572	8200	1500	2031	979	420	824	39	668
Patients with ≥1 TEAE	6365 (30.1)	3757 (28.2)	2250 (21.5)	1331 (47.0)	655 (24.4)	812 (45.3)	144 (25.5)	403 (32.8)	38 (49.4)	501 (42.9)
By MedDRA Preferred Term^*e*^:										
Headache	607 (2.9)	378 (2.8)	201 (1.9)	176 (6.2)	76 (2.8)	62 (3.5)	10 (1.8)	36 (2.9)	2 (2.6)	37 (3.2)
Hypothyroidism	598 (2.8)	430 (3.2)	224 (2.1)	206 (7.3)	31 (1.2)	75 (4.2)	5 (0.9)	23 (1.9)	—	34 (2.9)
Scoliosis	424 (2.0)	222 (1.7)	132 (1.3)	90 (3.2)	41 (1.5)	81 (4.5)	7 (1.2)	20 (1.6)	3 (3.9)	43 (3.7)
ADHD	390 (1.8)	216 (1.6)	157 (1.5)	58 (2.0)	75 (2.8)	37 (2.1)	4 (0.7)	23 (1.9)	2 (2.6)	31 (2.7)
Arthralgia	381 (1.8)	214 (1.6)	147 (1.4)	66 (2.3)	50 (1.9)	49 (2.7)	16 (2.8)	22 (1.8)	—	23 (2.0)
Secondary hypothyroidism	321 (1.5)	264 (2.0)	95 (0.9)	169 (6.0)	16 (0.6)	15 (0.8)	1 (0.2)	—	—	20 (1.7)
Precocious puberty	264 (1.2)	141 (1.1)	86 (0.8)	54 (1.9)	34 (1.3)	9 (0.5)	17 (3.0)	43 (3.5)	—	18 (1.5)
Otitis media	202 (1.0)	90 (0.7)	39 (0.4)	51 (1.8)	11 (0.4)	69 (3.9)	1 (0.2)	10 (0.8)	1 (1.3)	19 (1.6)
URTI	201 (1.0)	121 (0.9)	50 (0.5)	71 (2.5)	15 (0.6)	42 (2.3)	2 (0.4)	5 (0.4)	1 (1.3)	15 (1.3)

Abbreviations: ADHD, attention-deficit/hyperactivity disorder; MedDRA, Medical Dictionary for Regulatory Activities; N, number; URTI, upper respiratory tract infection.

^a^ Includes 335 patients with unknown diagnostic group.

^b^ Additional preferred terms with frequency ≥2.0% for OGHD [N (%)] are delayed puberty, 85 (3.0); adrenal insufficiency, 79 (2.8); hypogonadism, 74 (2.6); secondary hypogonadism, 70 (2.5); pyrexia, 66 (2.3); vomiting, 64 (2.3); hypopituitarism, 66 (2.3); constipation, 58 (2.0); and gastroenteritis, 58 (2.0).

^c^ Additional preferred terms with frequency ≥1.0% for TS [N (%)] are ovarian failure, 66 (3.7); melanocytic naevus, 62 (3.5); primary hypothyroidism, 41 (2.3); autoimmune thyroiditis, 34 (1.9); delayed puberty, 33 (1.8); ear infection, 26 (1.5); aortic dilation, 25 (1.4); hypogonadism, 25 (1.4); hypertension, 24 (1.3); bicuspid aortic valve, 21 (1.2); vitamin D deficiency, 20 (1.1); pharyngitis streptococcal, 19 (1.1); deafness, 18 (1.0); and vomiting, 18 (1.0).

^d^ Additional preferred terms with frequency ≥2.0% for CRI [N (%)] are renal transplant, 9 (11.7); anemia, 3 (3.9); chronic kidney disease, 3 (3.9); thrombocytopenia, 2 (2.6); urinary tract infection, 2 (2.6); bronchitis, 2 (2.6); hypertension, 2 (2.6); primary hypothyroidism, 2 (2.6); renal impairment, 2 (2.6); and vomiting, 2 (2.6).

^e^ Individual TEAEs are summarized by case, not by patient. A patient may have >1 TEAE.

### Mortality

At study closure, 51 deaths were reported; 45 occurred in 22,311 GH-treated patients and six in 457 untreated patients. On the basis of 42 deaths in 21,106 patients eligible for inclusion in the SMR calculation (mean ± SD follow-up of 4.3 ± 3.1 years), the crude mortality rate (95% CI) for all-cause mortality for GH-treated patients across all diagnoses was 0.46 (0.33, 0.62)/1000 PY, and the overall SMR (95% CI) was 0.61 (0.44, 0.82). The SMR calculation was repeated for patients who had ≥4 years of follow-up or who died at any time during the study (N = 9504), and the SMR was 0.81 (0.58, 1.10) with a mean follow-up of 7.1 ± 2.6 years. The only diagnostic subgroup with a statistically significantly elevated SMR was the OGHD group as a result of malignant neoplasm [14 deaths; SMRs of 5.87 (3.21, 9.85) overall and 7.16 (3.91, 12.01) for the 4-year follow-up population]. A comparison of mortality incidence in GeNeSIS using aggregated regional World Health Organization general population rates (with more granular pediatric age ranges) but with slightly lower SMRs were recently published (12).

### Diabetes and abnormal glucose metabolism

A total of 38 incident cases of diabetes mellitus (19 type 1, 18 type 2, and 1 case with type not specified) were reported in a cohort of 21,448 patients eligible for analysis, with mean ± SD follow-up from start of GH therapy of 5.0 ± 3.5 years (107,101 PY). Of the 18 reported cases of T2DM, two cases occurred within 1 year of start of GH therapy, nine cases between 1 and 4 years, and seven cases in patients with >4 years of GH therapy. In addition, there were 75 reported cases of other abnormal glucose metabolism conditions (including 27 reports of impaired glucose tolerance, nine reports of impaired fasting glucose, and cases of reported insulin resistance, hyperglycemia, and hyperinsulinemia). SIRs (95% CI) for all countries combined were calculated as 0.92 (0.56, 1.44) for type 1 diabetes mellitus and 3.77 (2.24, 5.96) for T2DM (Table 4). Because the SEARCH for Diabetes in Youth reference data are based on US patients only, SIRs were calculated separately for only patients from the United States: 1.08 (0.49, 2.05) for type 1 diabetes mellitus and 4.67 (2.13, 8.86) for T2DM (Supplemental Table 3). For T2DM, the statistically significantly elevated SIR observed for all diagnoses combined appeared to be driven by girls with TS [three cases; SIR (95% CI): 6.46 (1.33, 18.89)] and by patients with OGHD [eight cases; SIR (95% CI): 9.35 (4.04, 18.43)]. The SIR for T2DM was not statistically significantly elevated for other short stature diagnoses (Table 4); no cases of T2DM were observed during GeNeSIS in patients with ISS or SHOX-D. Risk factors for development of T2DM were reported for 13 of the 18 incident cases (Table 4).

**Table 4.** Incidence of Diabetes Mellitus in GH-Treated Patients for All Countries Combined

Diagnostic Group	N	PY	Diabetes Type	Cases^*a*^*^,^*^*b*^	Rate per 100,000 PY (95% CI)	Expected Cases	SIR (95% CI)
All	21,448^*c*^	107,101	Type 1	19	17.7 (10.7–27.7)	20.6	0.9 (0.6–1.4)
			Type 2	18	16.8 (10.0–26.6)	4.8	3.8 (2.2–6.0)
GHD	13,507^*d*^	68,526	Type 1	10	14.6 (7.0–26.8)	13.2	0.8 (0.4–1.4)
			Type 2	12^*e*^*^,^*^*f*^	17.5 (9.1–30.6)	3.1	3.9 (2.0–6.9)
IGHD	10,585	49,123	Type 1	4	8.1 (2.2–20.9)	9.4	0.4 (0.1–1.1)
			Type 2	4^*e*^	8.1 (2.2–20.9	2.2	1.8 (0.5–4.7)
OGHD	2874	19,211	Type 1	6	31.2 (11.5–68.0)	3.7	1.6 (0.6–3.5)
			Type 2	8^*f*^	41.6 (18.0–82.1)	0.9	9.4 (4.0–18.4)
TS	1815	10,423	Type 1	3	28.8 (5.9–84.1)	2.0	1.5 (0.3–4.4)
			Type 2	3^*g*^	28.8 (5.9–84.1)	0.5	6.5 (1.3–18.9)
ISS	2645	11,019	Type 1	3	27.2 (5.6–79.6)	2.1	1.4 (0.3–4.1)
			Type 2	0	0.0 (0.0–33.5)	0.5	0.0 (0.0–7.5)
SGA	1111	5689	Type 1	0	0.0 (0.0–64.9)	1.1	0.0 (0.0–3.4)
			Type 2	2^*h*^	35.2 (4.3–127.0)	0.3	7.9 (1.0–28.5
Other	1488	7491	Type 1	3	40.1 (8.3–117.0)	1.4	2.1 (0.4–6.1)
			Type 2	1^*i*^	13.4 (0.3–74.4)	0.3	3.0 (0.1–16.7)

Abbreviations: MELAS, mitochondrial encephalopathy, lactic acidosis, and stroke-like episodes; N, number.

^a^ An additional case was reported for which type of diabetes was not defined as type 1 or type 2.

^b^ An additional seven cases were reported in patients with known underlying pathology causative for diabetes (cystic fibrosis‒related diabetes, two cases; MELAS syndrome, two cases; and one case each of sideroblastic anemia, post pancreatic surgery, and steroid-induced diabetes); these events were not included as cases for the SIR calculation.

^c^ Includes patients with SHOX-D, CRI, and unknown short stature‒related diagnoses who had no cases of incident diabetes.

^d^ Includes patients for whom type of GHD has not been specified.

^e^ Includes one patient with IGHD and risk factor of history of obesity.

^f^ Includes patients with OGHD and the following risk factors: childhood cancer survivors with GHD due to leukemia and irradiation (three patients), history of craniopharyngioma and obesity (one patient), history of glioma and obesity (one patient), and preexisting insulin resistance (one patient with hypopituitarism due to *PROP1* gene defect).

^g^ Patients with TS considered at increased risk; one patient with preexisting impaired glucose tolerance.

^h^ Patients with Russell-Silver syndrome (both patients) considered at increased risk.

^i^ Patient with Prader-Willi syndrome and obesity.

### Primary cancers

Of the 20,187 patients without a history of malignancy who were eligible for analysis, 12,734 (63%) had GHD, of whom 10,413 (52%) had IGHD, 1804 (9%) had OGHD due to congenital GHD, and 467 (2%) had OGHD due to acquired causes (including craniopharyngioma and pilocytic astrocytoma). Other major diagnoses included ISS, 2579 (13%); TS, 1782 (9%); and being born SGA, 1222 (6%). Baseline and treatment characteristics of the cohort (all diagnoses combined) were similar to those of the overall safety cohort (data not shown).

From ∼89,000 PY of follow-up, 14 malignant primary neoplasms were identified, with a crude incidence rate estimated at 15.8/100,000 PY and a mean age at reported onset of cancer of 13.6 years. Of the 14 reported cases of primary cancer, three cases occurred within 1 year of start of GH therapy, five cases between 1 and 4 years, and six cases in patients with >4 years of GH therapy. The SIR (95% CI) for primary cancers in GH-treated patients was 0.71 (0.39, 1.20) for all countries combined; no individual country had a significantly elevated SIR (Table 5).

**Table 5.** Primary Cancer Cases and Standardized Incidence Ratios in Patients Without Cancer History for All Sites/Types of Cancer and Lymphomas Only

Country^*a*^	N	PY	Observed Cases	Expected Cases	SIR (95% CI)
All sites/types of cancer					
Canada	710	3671	3^*b*^	1.05	2.87 (0.59–8.38)
France	1544	7876	3^*c*^	2.16	1.39 (0.29–4.07)
Germany	2580	14,825	5^*d*^	4.03	1.24 (0.40–2.89)
Japan	2230	7302	1^*e*^	0.99	1.01 (0.03–5.63)
USA	8734	33,957	2^*f*^	6.14	0.33 (0.04–1.18)
Overall	20,146	88,749	14^*g*^*^,^*^*h*^	19.62	0.71 (0.39–1.20)
Lymphomas only					
France	1544	7876	1	0.24	4.21 (0.11–23.44)
Germany	2580	14,825	4	0.39	10.25 (2.79–26.25)
Overall	20,146	88,749	5	2.59	1.93 (0.63–4.51)

Abbreviation: N, number.

^a^ Countries with no incident cases are not listed in the table but are included in the overall SIR.

^b^ Ewing sarcoma, osteochondroma, and pancreatic neuroendocrine tumor.

^c^ Gonadoblastoma, T-cell lymphoma, and rectal adenocarcinoma.

^d^ B-cell lymphoma, Burkitt-like lymphoma, Burkitt lymphoma, lymphoma, and malignant schwannoma.

^e^ Germinoma.

^f^ Germ-cell tumor and skin cancer.

^g^ Specific risk factors reported: (i) recurrent neurofibromatosis and Gardner syndrome (case of rectal adenocarcinoma in a French patient, (ii) hamartomas and neurofibromatosis (pancreatic neuroendocrine tumor in a Canadian patient, (iii) pilocytic astrocytoma (malignant schwannoma in a German patient), and (iv) streak gonad of a French patient with 46,XY mixed gonadal dysgenesis (gonadoblastoma).

^h^ Three cases were in patients with syndromic short stature diagnoses (two lymphomas in German patients with Russell-Silver syndrome and TS and the Ewing sarcoma in a Canadian patient with TS).

Because lymphoma represented the most common tumor type, the SIR (95% CI) was calculated separately for lymphoma cases. The SIR for all countries combined was 1.93 (0.63, 4.51), but for Germany specifically, with four of the five cases, the SIR was statistically significantly elevated (Table 5). Four of the 14 patients had a defined predisposition to cancer (Table 5), whereas 10 of the affected patients had no recorded specific risk factors for malignancy; three of the cases were in patients with syndromic short stature diagnoses (Russell-Silver syndrome and TS for two of the lymphoma cases and TS for the Ewing sarcoma case).

### Neoplasm recurrences

There were 85 reports of recurrences in 74 of 1087 (6.8%) GH-treated children with at least one follow-up visit available and a history of previous neoplasm. Multiple recurrences were reported for seven patients: four with multiple craniopharyngioma recurrences (nine reported episodes), one with medulloblastoma recurrences (four episodes), one with optic glioma recurrences (three episodes), and one with ovarian fibroma recurrences (two episodes). There were 77 cases of intracranial tumor recurrences in 67 of 823 patients (8.1%), with craniopharyngioma recurrence being most common (42 episodes in 37 patients), followed by recurrence of astrocytoma (11 cases) and medulloblastoma (nine episodes in six patients). Although direct comparison between treatment groups is not appropriate because of inherent uncontrolled biases, for completeness of the record, there were nine reports of recurrences in nine of 148 untreated patients (6.1%), including four astrocytoma recurrences.

### SNs

Among 622 GH-treated survivors of childhood cancers, there were 34 reports of SNs in 31 patients (5.0%); 10 SNs were reported in nine of 114 untreated childhood cancer survivors (7.9%). Supplemental Table 4 lists details of all SNs recorded in GeNeSIS. As reported in the Childhood Cancer Survivor Study (CCSS) (17) and in a previously published analysis from GeNeSIS (22), the most common SNs in GH-treated patients are meningiomas. All four patients who developed a meningioma in the final GeNeSIS data set had reported cranial irradiation as part of their treatment of primary disease. Radiation therapy for the primary neoplasm was reported for 25 of the 31 GH-treated patients with any SN.

### Cerebrovascular disease

Sixteen patients were reported to have TEAEs of nontraumatic cerebrovascular disease during GeNeSIS (Table 2). Three intracranial hemorrhages were reported in GH-treated patients in the GeNeSIS database. The first was a fatal stroke described as “intracranial hemorrhage” after a renal transplant in a patient with CRI. The second was a case of “cerebral hemorrhage” in a patient with a history of optic glioma and many cerebrovascular anomalies. The third case was reported as “hemorrhage into glioma” in a patient with a history of glioma, astrocytoma, and tumor resection. Supplemental Table 5 provides details of short stature diagnosis and risk factors for all 16 cerebrovascular disease cases: 11 had a prior intracranial tumor, one had Ewing sarcoma, one was the previously described patient with CRI, and three patients had IGHD.

## Discussion

GeNeSIS was a large observational study of GH treatment outcomes in children with short stature and/or growth failure conducted over 16 years and enrolling 22,845 patients from 30 countries. Patient baseline characteristics were consistent with those of a broad population of pediatric GH users for age, sex, short stature diagnoses, and GH dose. Therefore, the results provide important real-world safety information from a large cohort.

The reported AE rate in the final GeNeSIS database (crude incidence of 175.3/1000 PY) appeared higher than in other observational studies of pediatric GH use, ranging from 5.6 to 103.1 per 1000 PY (23). However, GeNeSIS and the Pfizer International Growth Study were the only studies to require reporting of all AEs, irrespective of potential causality by GH. In agreement with findings from the National Cooperative Growth Study (NCGS) (2), the short stature diagnoses with the highest AE and SAE incidences were CRI and OGHD because of the severity of the underlying medical conditions.

During the 16-year duration of data collection in GeNeSIS, a number of potential safety issues related to the use of GH were raised and investigated. The French SAGhE study reported an increased mortality rate in young adults previously treated with GH during childhood for IGHD, ISS, and SGA (10). Although GeNeSIS is not directly comparable to SAGhE, especially in length of follow-up, the GeNeSIS data are reassuring, showing no increased mortality rate during the study in the same diagnostic groups, consistent with the SAGhE findings from Belgium, The Netherlands, and Sweden (24) and a recent assessment of mortality in Swedish GH-treated patients (25).

With >100,000 PY of GH exposure, 18 cases of T2DM were identified, representing an incidence rate of 16.8/100,000 PY. The final GeNeSIS incidence rate is lower than the previously reported 29.3/100,000 PY rate based on GeNeSIS data to September 2007 (13) and the 34.4/100,000 PY of GH treatment observed in the Pfizer International Growth Study (KIGS) >15 years ago (9). The incidence of T2DM across the whole study period was increased compared with general population rates: by 3.8-fold vs 6.5-fold in the earlier GeNeSIS analysis (14) and 6.4-fold in the KIGS analysis (9). Increased risk for diabetes appears largely confined to those with risk factors (26); in GeNeSIS, 13 of the 18 patients with incident cases of T2DM in the main analysis had risk factors for diabetes. In particular, patients with OGHD had the highest risk for T2DM when compared with the general population, including three cases with increased risk due to a history of leukemia irradiation (27) and one with concomitant Prader-Willi syndrome. In the absence of known diabetes risk factors, two recent studies exhibited no effect of GH on the rate of diabetes or negative effect on glucose homeostasis. The French SAGhE study data indicated no difference in the prevalence of diabetes between GH-treated patients with IGHD, ISS, or SGA and the general population (28). Although no deterioration in glucose homeostasis or new cases of diabetes were found in children with GHD in a 6-year follow-up study, a positive influence of GH/IGF-I on islet *β*-cell secretory capacity (29) was observed. This suggests that clinicians should focus efforts on lifestyle interventions in GH-treated patients with T2DM risk factors.

Concerns remain that GH treatment may be associated with primary cancer induction because of the mitogenic action of GH and high rates of specific cancer types in patients with acromegaly. In comparison with general population cancer registries, there appeared to be no higher risk for all-sites primary cancers in GH-treated patients in GeNeSIS, the same finding as in analyses of patients without risk factors for malignancy from both the KIGS and NCGS databases (2, 30). In addition, the crude incidence rate of 15.8/100,000 PY in GeNeSIS was similar to that from the KIGS: 16.4 cases/100,000 PY (30). A recent report from the pan-European SAGhE study (31) indicated no overall raised cancer-related morbidity or mortality risk in patients with growth failure without other major disease: SIR (95% CI) of 1.0 (0.6, 1.4) and SMR (95% CI) of 0.8 (0.4, 1.6). The authors concluded that their results did not generally support a carcinogenic effect of GH but the raised incidence of bone and bladder cancers in GH-treated patients and Hodgkin lymphoma with increasing follow-up required further investigation.

The most prevalent primary cancer type in GeNeSIS was lymphoma, with an elevated but not statistically significant overall SIR of 1.9 (0.6, 4.5). However, for Germany, with four of the five cases, the SIR was statistically significant at 10.3 (2.8, 26.3). Previously, an SMR of 11.5 (1.4, 41.3) and an SIR of 2.3 (0.3, 8.5) were reported for Hodgkin disease (two cases) in a cohort of 1848 cadaveric GH-treated patients (32). Of the GeNeSIS cases, at least three were non-Hodgkin type and the remaining two cases were unknown. The small number of cases hinders interpretation of such a finding, and there is no explanation for an increased risk in Germany, although two of the cases were in patients with syndromic short stature diagnoses. Two recent literature reviews found no association between GH therapy during childhood in children without prior cancer or known risk factors for developing cancer (8, 26). Patients with risk factors for cancer development were included in our analyses as long as they had not had previous cancer. The genotype/phenotype of such patients must be taken into account and the patients closely monitored for neoplastic disease during GH treatment.

The recurrence rate (crude incidence per 1000 PY) for intracranial tumors in GH-treated GeNeSIS patients was 8.1% (19.4%) overall, and 13.7% (29.9%) and 2.8% (8.5%) for craniopharyngioma and medulloblastoma, respectively. The GeNeSIS craniopharyngioma recurrence rate is broadly comparable to that in the literature, but rates of craniopharyngioma recurrence vary depending on the extent of surgical resection, ranging between 17% and 36% for gross total resection (8% to 10% when neuroradiologically confirmed) to between 43% and 67%, for partial resection (33). A retrospective study of more than 500 medulloblastoma survivors diagnosed and treated between 1980 and 1993 found no effect of GH treatment on risk of tumor recurrence (34). Analyses of GH-treated survivors of childhood medulloblastoma followed up in the NCGS determined a medulloblastoma recurrence rate of 7.2% (35), whereas the KIGS database reported 92% relapse-free survival at 4.6 years (36). A number of older retrospective studies (6, 35, 37) and more recent meta-analyses (38, 39) indicated that GH-treated patients with a history of intracranial tumor had lower rates of recurrence than untreated patients, but this likely reflects a selection bias, with those at a lower risk for tumor recurrence more likely to receive GH therapy. Nevertheless, according to data from many study cohorts, including GeNeSIS, there appears to be no evidence for increased risk of intracranial tumor recurrence in children treated with GH.

The final results from GeNeSIS do not provide any direct evidence supporting an increased risk of SNs in GeNeSIS. However, a previous analysis of GeNeSIS (12) concluded that the initial study findings were consistent with early reports from the CCSS, which indicated that GH therapy was associated with a higher relative risk of SNs (6, 7). At the close of GeNeSIS, the percentage of GH-treated survivors of childhood cancer who developed an SN was 5%, with a crude incidence of 12/1000 PY. When the analysis was restricted to only those patients with intracranial neoplasms, the percentage of patients affected was 1.3%, with a crude incidence of 3/1000 PY. Meningioma was among the most common SNs in GH-treated patients in GeNeSIS; however, only four cases were reported in three patients, and these represented all of the observed intracranial SNs. The low rate of intracranial SNs is therefore consistent with a subsequent CCSS follow-up report based on an extended length of surveillance, which did not show a statistically significantly higher risk of secondary central nervous system tumors among long-term survivors who had received GH-therapy during childhood (40).

With three cases of hemorrhagic stroke in patients with considerable risk factors, GeNeSIS data were not consistent with the finding of increased risk for intracranial hemorrhage in the French SAGhE cohort (11). Similarly, only one intracranial hemorrhage and two unspecified stroke cases were identified in 1024 adult patients with childhood-onset GHD who received GH as children and were subsequently followed up in the Hypopituitary Control and Complications Study; all three patients had underlying risk factors: two patients with a history of brain tumor, surgery, and radiotherapy and one with a history of meningocele surgery (41).

Our data should be interpreted in light of certain limitations. Patient recruitment was solely at the discretion of the investigator; although selection bias is a possibility, it may be mitigated by recruitment reflecting real-world prescribing for a large number of patients. In addition, because GeNeSIS was an observational study, reporting of cases was dependent on investigators from 30 countries, typically without sponsor monitoring of patient medical records. Although a potential underreporting of event cases must be considered, multiple data fields from the GeNeSIS and corporate pharmacovigilance databases were used to ascertain cases, and investigators were reminded of the importance of AE reporting throughout study participation. Similarly, GeNeSIS data were compared with general population registries from countries with varied quality of health care systems and may be subject to other biases. The average follow-up time in GeNeSIS was relatively short (mean, 4.2 years for GH-treated patients) for consideration of conditions such as T2DM and cancer and only during childhood GH treatment, with the possibility that a higher risk may be revealed with longer GH treatment and/or follow-up. However, it is noteworthy that ∼60% of both T2DM and primary cancer cases in GeNeSIS occurred within 4 years of the start of GH therapy. The period of follow-up marks a critical difference between GeNeSIS and analyses from the CCSS and SAGhE cohorts, where follow-up extended past the period of pediatric GH treatment; therefore, average follow-up per patient was much longer.

In conclusion, the results of the large GeNeSIS observational program indicate that the benefit-risk profile of GH remains favorable, although we acknowledge that average follow-up time within the study was relatively short. Compared with general population registries, GH-treated patients in GeNeSIS had no increased risk of early mortality, except patients with previous malignancy, and no increased risk for all-cause primary cancers. GH-treated patients in GeNeSIS did have an increased risk for T2DM, but most of these patients had diabetes risk factors. In addition, no cases of hemorrhagic stroke were observed in patients without significant risk factors. Although the GeNeSIS data are reassuring overall, specific safety findings emphasize the need to monitor GH-treated patients for abnormalities in glucose metabolism and those with a history of previous neoplasm and irradiation for development of subsequent neoplasms.

## Supplementary Material

Supplemental Datas

## Abbreviations:

AE: adverse event
CCSS: Childhood Cancer Survivor Study
CRI: chronic renal insufficiency
GeNeSIS: Genetics and Neuroendocrinology of Short Stature International Study
GHD: growth hormone deficiency
IGHD: idiopathic GHD
ISS: idiopathic short stature
KIGS: Pfizer International Growth Study
NCGS: National Cooperative Growth Study
OGHD: organic growth hormone deficiency
PY: person-year
SAE: serious AE
SAGhE: Safety and Appropriateness of Growth hormone treatments in Europe
SGA: small for gestational age
SHOX-D: short stature homeobox-containing gene deficiency
SIR: standardized incidence ratio
SMR: standardized mortality ratio
SN: second neoplasm
T2DM: type 2 diabetes mellitus
TEAE: treatment-emergent AE
TS: Turner syndrome
