# Supplementary material for: Safety Outcomes During Pediatric GH Therapy: Final Results From the Prospective GeNeSIS Observational Program
Source: J Clin Endocrinol Metab. 2018 Sep 13;104(2):379–89. doi: 10.1210/jc.2018-01189 (PMC6300411; doi:10.1210/jc.2018-01189)
Supplement: Supplemental Datas [file jc.2018-01189.sd1.docx]

Supplementary Table 1: Investigative sites and patient numbers by country.

| **Country** | **Number of sites** | **All patients n (%)** | **GH-treated n (%)** | **Untreated n (%)** | **Unknown n (%)** |
| --- | --- | --- | --- | --- | --- |
| Total | 827 | 22845 (100.0) | 22311 (100.0) | 457 (100.0) | 77 (100.0) |
| Australia | 7 | 87 (0.4) | 87 (0.4) | 0 (0.0) | 0 (0.0) |
| Austria | 8 | 103 (0.5) | 103 (0.5) | 0 (0.0) | 0 (0.0) |
| Belgium | 10 | 83 (0.4) | 83 (0.4) | 0 (0.0) | 0 (0.0) |
| Canada | 14 | 870 (3.8) | 850 (3.8) | 17 (3.7) | 3 (3.9) |
| Czech Republic | 15 | 344 (1.5) | 332 (1.5) | 12 (2.6) | 0 (0.0) |
| Denmark | 3 | 6 (0.0) | 5 (0.0) | 0 (0.0) | 1 (1.3) |
| Finland | 6 | 28 (0.1) | 28 (0.1) | 0 (0.0) | 0 (0.0) |
| France | 74 | 1821 (8.0) | 1667 (7.5) | 150 (32.8) | 4 (5.2) |
| Germany | 151 | 2723 (11.9) | 2685 (12.0) | 24 (5.3) | 14 (18.2) |
| Greece | 8 | 305 (1.3) | 290 (1.3) | 14 (3.1) | 1 (1.3) |
| Hungary | 13 | 334 (1.5) | 332 (1.5) | 2 (0.4) | 0 (0.0) |
| Iceland | 1 | 48 (0.2) | 48 (0.2) | 0 (0.0) | 0 (0.0) |
| India | 12 | 242 (1.1) | 212 (1.0) | 30 (6.6) | 0 (0.0) |
| Italy | 62 | 788 (3.4) | 771 (3.5) | 13 (2.8) | 4 (5.2) |
| Japan | 190 | 2356 (10.3) | 2352 (10.5) | 0 (0.0) | 4 (5.2) |
| Kazakstan | 1 | 29 (0.1) | 29 (0.1) | 0 (0.0) | 0 (0.0) |
| Lithuania | 1 | 90 (0.4) | 90 (0.4) | 0 (0.0) | 0 (0.0) |
| Netherlands | 1 | 320 (1.4) | 317 (1.4) | 0 (0.0) | 3 (3.9) |
| Norway | 3 | 8 (0.0) | 8 (0.0) | 0 (0.0) | 0 (0.0) |
| Pakistan | 11 | 178 (0.8) | 172 (0.8) | 6 (1.3) | 0 (0.0) |
| Russia | 1 | 195 (0.9) | 180 (0.8) | 15 (3.3) | 0 (0.0) |
| Singapore | 1 | 16 (0.1) | 13 (0.1) | 3 (0.7) | 0 (0.0) |
| Slovakia | 4 | 94 (0.4) | 94 (0.4) | 0 (0.0) | 0 (0.0) |
| South Africa | 5 | 109 (0.5) | 106 (0.5) | 1 (0.2) | 2 (2.6) |
| Spain | 56 | 1422 (6.2) | 1404 (6.3) | 9 (2.0) | 9 (11.7) |
| Sweden | 6 | 53 (0.2) | 53 (0.2) | 0 (0.0) | 0 (0.0) |
| Taiwan | 4 | 115 (0.5) | 114 (0.5) | 0 (0.0) | 1 (1.3) |
| Thailand | 1 | 33 (0.1) | 33 (0.1) | 0 (0.0) | 0 (0.0) |
| United Kingdom | 5 | 42 (0.2) | 41 (0.2) | 1 (0.2) | 0 (0.0) |
| United States | 154 | 10003 (43.8) | 9812 (44.0) | 160 (35.0) | 31 (40.3) |

Supplementary Table 2: Frequency of serious adverse events by MedDRA preferred term in ≥0.04% of all GH-treated patients, split by main short stature diagnosis.

|  | **All^a^** | **GHD^b^** | **IGHD** | **OGHD^c^** | **ISS** | **TS^d^** | **SHOX-D** | **SGA** | **CRI^e^** | **Other^f^** |
| --- | --- | --- | --- | --- | --- | --- | --- | --- | --- | --- |
| **N (%)** | **22294** | **14039** | **11032** | **2957** | **2843** | **1868** | **575** | **1276** | **87** | **1226** |
| **Patients with no SAE** | **21727** | **13689** | **10901** | **2740** | **2820** | **1802** | **560** | **1250** | **76** | **1153** |
| **Patients with at least one SAE** | **567 (2.54)** | **350 (2.49)** | **131 (1.19)** | **217 (7.34)** | **23 (0.81)** | **66 (3.53)** | **15 (2.61)** | **26 (2.04)** | **11 ( 12.64)** | **73 (5.95)** |
| By MedDRA Preferred Term^g^: |  |  |  |  |  |  |  |  |  |  |
| Pneumonia | 24 (0.11) | 12 (0.09) | 5 (0.05) | 7 (0.24) | - | 4 (0.21) | - | - | 1 (1.15) | 7 (0.57) |
| Craniopharyngioma | 20 (0.09) | 19 (0.14) | 2 (0.02) | 17 (0.57) | - | - | - | - | - | - |
| Hypoglycaemia | 19 (0.09) | 19 (0.14) | 4 (0.04) | 15 (0.51) | - | - | - | - | - | - |
| Gastroenteritis | 17 (0.08) | 12 (0.09) | - | 12 (0.41) | - | 2 (0.11) | - | - | 1 (1.15) | 2 (0.16) |
| Vomiting | 16 (0.07) | 9 (0.06) | 2 (0.02) | 7 (0.24) | - | 3 (0.16) | - | - | 1 (1.15) | 3 (0.24) |
| Seizure | 16 (0.07) | 12 (0.09) | 5 (0.05) | 7 (0.24) | - | 2 (0.11) | - | - | - | 2 (0.16) |
| Appendicitis | 14 (0.06) | 8 (0.06) | 5 (0.05) | 2 (0.07) | 1 (0.04) | 1 (0.05) | 3 (0.52) | - | - | 1 (0.08) |
| Scoliosis | 11 (0.05) | 5 (0.04) | 2 (0.02) | 3 (0.10) | 2 (0.07) | 1 (0.05) | - | 1 (0.08) | 1 (1.15) | 1 (0.08) |
| Epiphysiolysis | 11 (0.05) | 7 (0.05) | 3 (0.03) | 3 (0.10) | - | 3 (0.16) | - | - | - | 1 (0.08) |
| Adrenocortical insufficiency acute | 10 (0.04) | 10 (0.07) | 1 (0.01) | 9 (0.30) | - | - | - | - | - | - |
| Viral infection | 9 (0.04) | 8 (0.06) | 1 (0.01) | 7 (0.24) | - | - | - | - | - | 1 (0.08) |

Abbreviations: CRI, chronic renal insufficiency; GHD, growth hormone deficiency; IGHD, idiopathic GHD; ISS, idiopathic short stature; MedDRA, Medical Dictionary for Regulatory Activities; OGHD, organic GHD; SAE, serious adverse event; SGA, small for gestational age; SHOX-D, SHOX deficiency; TS, Turner syndrome.

^a^Includes 380 patients with unknown diagnostic group.

^b^Additional preferred terms with frequency ≥0.04% for GHD are headache [N (%)] 8 (0.06), influenza 7 (0.05), and dehydration 6 (0.04).

^c^Additional preferred terms with frequency ≥0.15% for OGHD are headache [N (%)] 7 (0.24), adrenal insufficiency 5 (0.17), pyrexia 5 (0.17), influenza 5 (0.17), and dehydration 5 (0.17).

^d^Additional preferred term with frequency ≥0.15% for TS is tonsillar hypertrophy [N (%)] 3 (0.16).

^e^Additional preferred terms with frequency ≥2.0% for CRI are chronic kidney disease [N (%)] 3 (3.45), urinary tract infection 2 (2.30), and renal transplant 2 (2.30).

^f^Additional preferred term with frequency ≥0.20% for Other is pyelonephritis [N (%)] 3 (0.24).

^g^Individual SAEs are summarized by case, not by patient. A patient may have >1 SAE.

Supplementary Table 3: Incidence of diabetes mellitus in US GH-treated patients.

| **Diagnostic group** | **N** | **Person-Years (PY)** | **Diabetes type** | **Cases^b,c^** | **Rate per 100,000 PY (95% CI)** | **Expected cases** | **SIR (95% CI)** |
| --- | --- | --- | --- | --- | --- | --- | --- |
|  |  |  |  |  |  |  |  |
| All | 9456^a^ | 43320 | Type 1 | 9 | 20.8 (9.5-39.4) | 8.3 | 1.1 (0.5-2.1) |
|  |  |  | Type 2 | 9 | 20.8 (9.5-39.4) | 1.9 | 4.7 (2.1-8.9) |
| GHD | 5030^d^ | 23515 | Type 1 | 4 | 17.0 (4.6-43.6) | 4.5 | 0.9 (0.2-2.3) |
|  |  |  | Type 2 | 6 | 25.5 (9.4-55.5) | 1.1 | 5.7 (2.1-12.5) |
| IGHD | 3953 | 16495 | Type 1 | 3 | 18.2 (3.8-53.2) | 3.2 | 1.0 (0.2-2.8) |
|  |  |  | Type 2 | 2 | 12.1 (1.5-43.8) | 0.7 | 2.7 (0.3-9.8) |
| OGHD | 1047 | 6879 | Type 1 | 1 | 14.5 (0.4-81.0) | 1.3 | 0.8 (0.0-4.2) |
|  |  |  | Type 2 | 4 | 58.2 (15.8-148.9) | 0.3 | 13.1 (3.6-33.4) |
| TS | 721 | 4003 | Type 1 | 1 | 25.0 (0.6-139.2) | 0.8 | 1.3 (0.0-7.3) |
|  |  |  | Type 2 | 1 | 25.0 (0.6-139.2) | 0.2 | 5.6 (0.1-31.3) |
| ISS | 2455 | 10092 | Type 1 | 3 | 29.7 (6.1-86.9) | 1.9 | 1.6 (0.3-4.5) |
|  |  |  | Type 2 | 0 | 0.0 (0.0-36.6) | 0.5 | 0.0 (0.0-8.2) |
| SGA | 298 | 1393 | Type 1 | 0 | 0.00 (0.0-264.9) | 0.3 | 0.0 (0.0-13.8) |
|  |  |  | Type 2 | 1 | 71.8 (1.8-400.1) | 0.1 | 16.1 (0.4-89.9) |
| Other | 702 | 3379 | Type 1 | 1 | 29.6 (0.8-164.9) | 0.7 | 1.5 (0.0-8.6) |
|  |  |  | Type 2 | 1 | 29.6 (0.8-164.9) | 0.2 | 6.7 (0.2-37.0) |

Abbreviations: CI, confidence interval; CRI, chronic renal insufficiency; GHD, growth hormone deficiency; IGHD, idiopathic GHD; ISS, idiopathic short stature; OGHD, organic GHD; SGA, small for gestational age; SHOX-D, SHOX deficiency; SIR, standardized incidence ratio; TS, Turner syndrome.

^a^Includes patients with SHOX-D, CRI, and unknown short stature-related diagnoses who had no cases of incident diabetes.

^b^An additional case was reported where type of diabetes was not defined as type 1 and type 2.

^c^An additional 2 cases were reported in patients with cystic fibrosis – known underlying pathology causative for diabetes; these events were not included as cases for the SIR calculation.

^d^Includes patients where type of GHD has not been specified.

Supplementary Table 4: Second neoplasms in childhood cancer survivors.

| **Patient** | **Primary Neoplasm/Other Diagnoses** | **Radiation^a^** | **Second Neoplasm(s)** |
| --- | --- | --- | --- |
| ***GH-treated patients*** | | | |
| 1 | Astrocytoma | Yes | Fallopian tube cyst |
| 2 | Ependymoma with recurrence | Yes | Osteosarcoma of skull |
| 3 | Glioma | - | Osteoma |
| 4 | Medulloblastoma | Yes | Acute lymphocytic leukemia |
| 5 | Medulloblastoma | Yes | Acute myeloid leukemia^b^ |
| 6 | Medulloblastoma | Yes | Myelodysplastic syndrome |
| 7 | Medulloblastoma | Yes | Pleomorphic xanthomatous astrocytoma |
| 8 | Medulloblastoma | Yes | Low-grade glioma^c^ |
| 9 | Medulloblastoma | Yes | Meningioma |
| 10 | Medulloblastoma | Yes | 1) Meningioma (left parietal), 2) Meningioma (right posterior occipital) |
| 11 | Medulloblastoma, Gorlin syndrome | Yes | 1) Meningioma -sphenoid wing, 2) Basal cell carcinoma, 3) Benign soft tissue lesion |
| 12 | Medulloblastoma | - | Benign bone lesion |
| 13 | Medulloblastoma | Yes | Bladder myoepithelioma |
| 14 | Medulloblastoma | Yes | Granular cell tumor (lingual) |
| 15 | Medulloblastoma | - | Ovarian stromal tumor |
| 16 | Medulloblastoma | Yes | Papillary thyroid cancer |
| 17 | Medulloblastoma | Yes | Spinal cord neoplasm^d^ |
| 18 | Medulloblastoma | Yes | Spinal tumor |
| 19 | Medulloblastoma | Yes | Uterine fibroid |
| 20 | Neuroblastoma | Yes | Osteochondroma |
| 21 | Neuroblastoma | - | Osteochondroma |
| 22 | Neuroblastoma | Yes | Pheochromocytoma |
| 23 | Neuroblastoma | Yes | Soft tissue sarcoma |
| 24 | Neuroblastoma | - | Thyroid nodule |
| 25 | Leukemia | Yes | Bone cyst (giant cell tumor of tendon) |
| 26 | Leukemia with CNS disease | Yes | Cerebral neuroblastoma^b^ |
| 27 | Leukemia | Yes | Osteochondroma |
| 28 | Leukemia | Yes | Osteochondroma |
| 29 | Retinoblastoma | Yes | Hurthle cell thyroid adenoma |
| 30 | Rhabdomyosarcoma | Yes | Meningioma |
| 31 | Rhabdomyosarcoma | - | Kaposi’s sarcoma (tongue) |
| ***Untreated patients*** | | | |
| 1 | Atypical teratoid/rhabdoid tumor | Yes | Meningeal sarcoma |
| 2 | Glioma, neurofibromatosis | - | Bone fibroma |
| 3 | Medulloblastoma | - | Renal cell carcinoma |
| 4 | Neuroblastoma | - | Follicular thyroid cancer |
| 5 | PNET/Ewing sarcoma | - | Osteochondroma |
| 6 | Leukemia | - | 1) Fibrous histiocytoma, 2) Osteochondroma |
| 7 | Leukemia | - | Fibrous histiocytoma |
| 8 | Leukemia | - | Osteochondroma |
| 9 | Leukemia | - | Meningioma |

Abbreviation: CNS, central nervous system; GH, growth hormone; MRI, magnetic resonance imaging; PNET, primitive neuroectodermal tumor

^a^Radiation therapy for primary cancers as reported in GeNeSIS diagnosis and Neoplasia Sub-study modules or in serious adverse event reports; a dash indicates no radiation reported in databases.

^b^Second malignancy resulted in death of patient.

^c^MRI indicated possible tumor recurrence or scar tissue. Subsequent MRI showed no evidence of residual tumor in the posterior fossa, but a stable lesion in the right basal ganglia which likely represented low-grade glioma.

^d^The reported spinal cord neoplasm observed on MRI was not confirmed on repeated imaging.

Supplementary Table 5: Cases of incident cerebrovascular disease and associated risk factors in GH-treated patients.^a^

| **Country** | **Short stature diagnosis** | **Cerebrovascular event  (as reported)** | **Additional risk factors/information** |
| --- | --- | --- | --- |
| ***Hemorrhagic disease*** | | | |
| Canada | OGHD | Cerebral hemorrhage | Optic glioma, NF, cranial irradiation, right Sylvian artery aneurysm, Circle of Willis arterial stenosis, carotid artery stenosis, cerebral arteritis, Moyamoya disease |
| USA | OGHD | Hemorrhage into glioma | Disseminated glioma/astrocytoma, no GH treatment at time of event |
| Hungary | CRI | Fatal event of stroke | Post renal transplant |
| ***Ischemic disease*** | | | |
| Canada | OGHD | Ischemic stroke | Medulloblastoma, Gorlin syndrome, CNS surgery |
| USA | OGHD | Right pontine infarct, right cerebellar infarct | Medulloblastoma, cranial irradiation |
| Germany | OGHD | Moyamoya disease^b^ | Pilocytic astrocytoma with progression, cranial irradiation |
| Canada | OGHD | Moyamoya disease^b^ | Medulloblastoma, cranial irradiation, CNS surgery |
| Canada | OGHD | Moyamoya disease^b^ | Germinoma, cranial/spinal irradiation, CNS surgery, chemotherapy |
| USA | OGHD | Moyamoya disease, TIA (x2) | Ewing sarcoma |
| USA | OGHD | TIA | Astrocytoma, cranial irradiation, CNS surgery |
| USA | OGHD | Possible TIA | Ependymoma, cranial/spinal irradiation, chemotherapy |
| Germany | IGHD | Occlusion of the vertebral artery |  |
| Japan | IGHD | Moyamoya disease^b^ |  |
| ***Unknown type*** | | | |
| Germany | OGHD | Cerebral attack | Craniopharyngioma, CNS surgery |
| Canada | OGHD | Cerebral subcortical lesion | Medulloblastoma, cranial irradiation and CNS surgery |
| Austria | IGHD | Cerebral attacks generalized | Receiving anticonvulsant therapy and had mental retardation. May represent epilepsy, rather than MedDRA coding of “cerebrovascular accident” |

Abbreviations: CNS, central nervous system; CRI, chronic renal insufficiency; GH, growth hormone; IGHD, idiopathic growth hormone deficiency; MedDRA, Medical Dictionary for Regulatory Activities; NF, neurofibromatosis; OGHD, organic growth hormone deficiency; SGA, small for gestational age; TIA, transient ischemic attack.

^a^In addition, 1 patient, included in the SGA diagnostic group, had MELAS syndrome and suffered from recurrent stroke-like episodes (synonymous with MELAS) that led to a fatal brain stem infarction. This case was not included in the overall case counts presented in Table 2 or this supplementary data.

^b^Moyamoya disease is a progressive occlusive disease of the cerebral vasculature with particular involvement of the Circle of Willis and the arteries that feed it and has been reported as a consequence of cranial irradiation (Scott RM, Smith ER. Moyamoya disease and Moyamoya syndrome. *New England Journal of Medicine* 2009 **360** 1226–1237.)

Supplementary Figure 1: Scheme to priortize evidence of abnormal glucose metabolism and diabetes cases and histories.

**Weak Evidence:**

Concomitant medication^b^

**Moderate Evidence:**

Specific diabetes case/history checkbox at each visit

**Strong Evidence:**

- Adverse event/pre-existing condition
- Serious adverse event
- Questionnaire response^a^

If medication is only potential reported evidence

Not considered as case

If checkbox is only potential reported evidence

Included in sensitivity analysis

Any combination of evidence including ≥1 of above

Included in main analysis

2 checkbox only T1DM cases

3 checkbox only T2DM cases

**Main analysis**

19 T1DM incident cases

SIR (95%CI) 0.92 (0.56-1.44)

18 T2DM incident cases

SIR (95%CI) 3.77 (2.24-5.96)

**Sensitivity analysis^c^**

21 T1DM incident cases

SIR (95%CI) 1.02 (0.63-1.56)

21 T2DM incident cases

SIR (95%CI) 4.40 (2.73-6.73)

Abbreviations: CI, confidence interval; SIR, standardized incidence ratio; T1DM, type 1 diabetes mellitus, T2DM, type 2 diabetes mellitus.

^a^Questionnaire to collect additional abnormal glucose metabolism case information was sent for cases reported to September 2007 (see Child et al 2011) but not for new reports after September 2007.

^b^Medications typically associated with treatment of hyperglycemia.

^c^Sensitivity analysis results not reported in main paper.

Reference: Child CJ, Zimmermann AG, Scott RS, Cutler GB Jr, Battelino T, Blum WF; for the GeNeSIS International Advisory Board. Prevalence and incidence of diabetes mellitus in GH-treated children and adolescents: analysis from the GeNeSIS observational research program. *J Clinical Endocrinol Metab.* 2011;96(6):E1025–E1034.
